# Divergent Trajectories of the Burden of MASLD Attributable to High Fasting Plasma Glucose in China and Globally: A GBD Study 1990–2021 and Projections to 2046

**DOI:** 10.1155/grp/1090542

**Published:** 2026-06-20

**Authors:** Xun Wu, Wei-Wei Zhang, Bo-Hu Wang, Yong-Feng Tang

**Affiliations:** ^1^ Hospital Office, Jiangbin Hospital of Guangxi Zhuang Autonomous Region, Nanning, Guangxi Province, China; ^2^ Department of Gastroenterology, The First People’s Hospital of Nanning, Nanning, Guangxi Province, China; ^3^ Information Network Center, Ruikang Hospital Affiliated to Guangxi University of Traditional Chinese Medicine, Nanning, Guangxi Province, China, gxtcm.com

**Keywords:** disability-adjusted life years, global burden of disease, high fasting plasma glucose, metabolic dysfunction–associated steatotic liver disease, projection, temporal trends

## Abstract

**Background:**

Metabolic dysfunction–associated steatotic liver disease (MASLD) is a growing global health concern. High fasting plasma glucose (HFPG) is a major metabolic driver, yet its long‐term burden and future trends in China versus globally remain unclear.

**Methods:**

Data from the Global Burden of Disease Study 2021 were analyzed. Deaths and disability‐adjusted life years (DALYs) were assessed using counts and age‐standardized rates (ASRs). Temporal trends were evaluated using estimated annual percentage change (EAPC). An age–period–cohort (APC) model was used to project the burden through 2046.

**Results:**

In 2021, HFPG‐attributable MASLD caused 10,021 deaths and 213,481 DALYs worldwide. From 1990 to 2021, global age‐standardized death and DALY rates increased significantly (EAPC, 2.22% and 2.02%, respectively). The burden was higher in males and increased with age, peaking in those aged ≥ 80 years. Projections suggest that absolute deaths and DALYs will continue to rise globally and in China through 2046, whereas ASRs are expected to decline, particularly among females. China showed a lower increase in age‐standardized death rate (EAPC, 1.28%) compared with the global average.

**Conclusion:**

The burden of HFPG‐attributable MASLD continues to increase globally and remains substantial in China. Although ASRs may stabilize or decline, population aging is expected to sustain growth in the absolute burden. These findings highlight the need for early metabolic screening, integrated management of metabolic and liver diseases, and targeted interventions for older adults and other high‐risk populations.

## 1. Introduction

Metabolic dysfunction–associated steatotic liver disease (MASLD), previously referred to as nonalcoholic fatty liver disease (NAFLD), has become the most prevalent chronic liver disease globally, affecting approximately 30%–40% of adults [1]. The 2023 international multisociety consensus updated the terminology to MASLD to highlight the central role of metabolic dysfunction in the disease’s pathogenesis [2]. MASLD represents a spectrum ranging from simple steatosis to metabolic dysfunction–associated steatohepatitis (MASH), which can progress to cirrhosis and hepatocellular carcinoma (HCC) [3]. Beyond liver‐related morbidity, MASLD is associated with significant extrahepatic systemic risks, with cardiovascular disease (CVD) frequently identified as a primary cause of mortality in this population [4].

Among the modifiable risk factors influencing the progression of MASLD, high fasting plasma glucose (HFPG) is a critical driver. HFPG serves as a biochemical manifestation of insulin resistance (IR), which promotes hepatic de novo lipogenesis (DNL) and lipid accumulation, thereby accelerating the transition to advanced fibrosis [5, 6]. Clinical evidence suggests a bidirectional relationship between glycemic abnormalities and liver health; approximately 70% of patients with Type 2 diabetes mellitus (T2DM) have comorbid MASLD, and T2DM is an independent risk factor for liver cirrhosis and HCC [7].

Despite the recognized link between hyperglycemia and MASLD, the “preventable burden” associated with HFPG has not been fully characterized within the theoretical minimum risk exposure level (TMREL) framework [8]. While global trends in MASLD have been explored, there is a lack of attribution analysis specifically focusing on HFPG, particularly in rapidly developing economies like China [9]. China faces a significant epidemiological transition where metabolic disorders are rising alongside an aging population [10]. Quantifying the specific contribution of HFPG to the MASLD burden in this context is important for informing public health policies and optimizing healthcare resources.

In this study, we utilized data from the Global Burden of Disease Study 2021 (GBD 2021) to systematically analyze MASLD‐related deaths and disability‐adjusted life years (DALYs) attributable to HFPG from 1990 to 2021. Although GBD 2021 continues to categorize this disease under the historical term NAFLD, we adopted the updated MASLD nomenclature, as previous studies have demonstrated a 95%–99% epidemiological overlap between the two populations [11] [12]. Consequently, the NAFLD category in the GBD framework was considered a reliable proxy for MASLD in this analysis.

We hypothesized that the global and national burden of MASLD attributable to HFPG has increased significantly over the past three decades and will continue to escalate through 2046, driven largely by aging and metabolic shifts. By applying age–period–cohort (APC) modeling to GBD data, this study is aimed at disentangling the drivers of these trends and providing evidence to support integrated metabolic and hepatic prevention strategies globally and in China.

## 2. Materials and Methods

### 2.1. Data Sources and Attribution Framework

Data for this study were retrieved from the GBD 2021 study, which provides a comprehensive and systematic assessment of the disease burden linked to 371 diseases and injuries and 88 risk factors from 1990 to 2021 across 204 countries and territories. The Global Health Data Exchange (GHDx) query tool (https://ghdx.healthdata.org/) was used to obtain all relevant data. This study was conducted following the GBD data use policy and complied with the relevant ethical standards and reporting guidelines for the secondary analysis of public databases [13].

The disease burden of MASLD (formerly NAFLD) was examined. Because GBD 2021 still categorizes this disease entity under the historical terminology of NAFLD, the present study adopted the updated nomenclature “MASLD” according to the 2023 international consensus definition. In this context, MASLD was considered epidemiologically comparable to the NAFLD category used in the GBD framework, as previous studies have demonstrated substantial overlap between the two populations [5, 14]. Within the GBD framework, the estimation of disease burden related to HFPG is based on the comparative risk assessment (CRA) methodology. HFPG exposure was operationalized as fasting plasma glucose levels exceeding the TMREL. This approach integrates population exposure distributions with relative risks (RRs) derived from meta‐analyses of epidemiological studies to calculate population attributable fractions (PAFs). These PAFs indicate the proportion of the disease burden that could be reduced if HFPG exposure were lowered to the TMREL. The TMREL refers to the level of exposure associated with the lowest theoretical disease risk. In the GBD 2021 report, the TMREL for fasting plasma glucose is defined as a distribution with a mean ranging from 4.8 to 5.4 mmol/L [15]. HFPG exposure levels were estimated using a Bayesian metaregression model (DisMod‐MR 2.1), which accounts for spatial and temporal variability across diverse data sources. All estimates were reported with 95% uncertainty intervals (UIs), representing the 2.5th and 97.5th percentiles of the posterior distribution, thereby reflecting uncertainties arising from data sources, model specifications, and the process of estimation [16].

### 2.2. Statistical Analysis and Trend Assessment

Disease burden was assessed using absolute numbers and age‐standardized rates (ASRs) of DALYs. The ASRs were calculated through the direct standardization method based on the GBD world standard population and expressed per 100,000 individuals to ensure comparability across different populations and time periods [17].

A linear regression model was fitted to the natural logarithm of the annual ASRs to analyze temporal trends in disease burden from 1990 to 2021: ln(ASR) = *α* + *β* *x* + *ε*, where *x* represents the calendar year and *β* denotes the regression coefficient. Before estimated annual percentage change (EAPC) estimation, log‐transformed ASR trends were visually inspected to confirm approximate linearity over time. The EAPC was further calculated using the following formula: EAPC = (exp(*β*) − 1) × 100*%*. The 95% confidence interval (CI) for the EAPC, derived from the regression model, categorized trends as follows: A significant increasing trend occurred when both the EAPC and the lower limit of its 95% CI were > 0; a significant decreasing trend occurred when both the EAPC and the upper limit of its 95% CI were < 0; otherwise, the trend was considered stable [18]. All statistical analyses and visualizations were conducted using R software (Version 4.3.2; R Foundation for Statistical Computing, Vienna, Austria). Major packages included “ggplot2,” “dplyr,” “Epi,” and “Nordpred.”

### 2.3. Forecasting Model

To assess future trends, we used the APC model to forecast the HFPG‐attributable MASLD burden from 2022 to 2046. This model characterizes the dynamic evolution of disease burden by decomposing age effects (indicating biological aging), period effects (reflecting medical advancements and macroenvironmental shifts), and cohort effects (signifying cumulative exposure differences across birth generations). The intrinsic estimator (IE) method was employed for parameter estimation to address the inherent nonidentifiability problem in the APC model [19]. The APC projections were generated under the assumption that historical temporal patterns, demographic transitions, and exposure trends would remain relatively continuous during the forecast period. Forecasts were integrated with GBD population projection data to evaluate future changes in disease burden under varying age, period, and cohort effects. Because long‐term forecasting is inherently associated with uncertainty, the projected estimates should be interpreted as trend‐based projections rather than precise predictions. In addition, the current GBD CRA framework enables quantitative attribution specifically for HFPG; therefore, other metabolic risk factors were not included in the present forecasting analyses.

### 2.4. Statistical Software

All statistical analyses were performed using R software (Version 4.3.2; R Foundation for Statistical Computing, Vienna, Austria). Major packages included “ggplot2,” “Epi,” and “Nordpred” for data visualization and APC‐related analyses. The GBD‐derived datasets analyzed during the current study and the corresponding cleaned datasets are available in the Supporting Information.

## 3. Results

### 3.1. Current Status and Comparative Analysis of HFPG‐Attributable MASLD Burden in China and Globally in 2021

#### 3.1.1. Overall Disease Burden Profile

**Figure 1:**
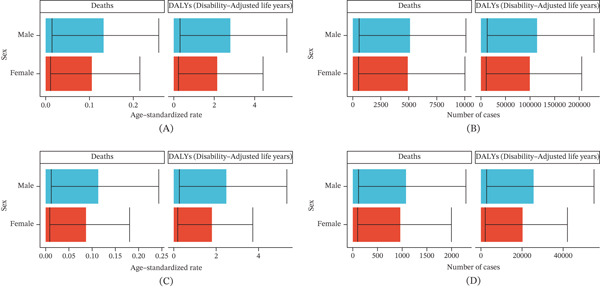
Numbers and ASRs of metabolic dysfunction–associated steatotic liver disease attributable to high fasting plasma glucose‐related deaths and DALYs for both sexes in China and globally in 2021. Abbreviations: DALYs, disability‐adjusted life years; ASRs, age‐standardized rates.

In 2021, MASLD attributable to HFPG accounted for a substantial health loss globally. The number of deaths reached 10,021 (95% UI: 1097–19,980), while DALYs totaled 213,481 (95% UI: 23,797–432,139). The age‐standardized death rate (ASDR) and age‐standardized DALY rate (ASDAR) were reported as 0.12 and 2.45 per 100,000 population, respectively (Tables 1 and 2). During the same period, China documented 2038 deaths (95% UI: 220–4105) and 45,832 DALYs (95% UI: 4935–92,455), representing 20.3% and 21.5% of the global burden, respectively. China’s ASDR (0.10 per 100,000) and ASDAR (2.13 per 100,000) were slightly below the global averages (Figure 1 and Tables 1 and 2). Overall, both global and Chinese estimates demonstrated a considerable burden of HFPG‐attributable MASLD, while China showed slightly lower ASRs than the global level.

**Table 1:** Burden of metabolic dysfunction–associated steatotic liver disease attributable to high fasting plasma glucose in China and globally: Death counts and age‐standardized death rates in 1990 and 2021, with temporal trends (1990–2021).

	Number of death cases (95% UI) in 1990	The age‐standardized death rate/100,000 (95% UI) in 1990	Number of death cases (95% UI) in 2021	The age‐standardized death rate/100,000 (95% UI) in 2021	EAPC (95% CI)
Global	2319 (247–4801)	0.06 (0.01–0.13)	10021 (1097–19,980)	0.12 (0.01–0.23)	2.22 (2.07–2.36)
Sex globally					
Female	1186 (126–2407)	0.06 (0.01–0.12)	4910 (519–10,022)	0.11 (0.01–0.21)	2.16 (2.03–2.29)
Male	1133 (122–2372)	0.07 (0.01–0.14)	5111 (566–10,107)	0.13 (0.01–0.26)	2.27 (2.11–2.42)
Age groups globally (years)					
25–29	3 (0–7)	0 (0–0)	6 (1–15)	0 (0–0)	1.11 (0.82–1.41)
30–34	6 (1–14)	0 (0–0)	15 (1–34)	0 (0–0.01)	1.11 (0.81–1.42)
35–39	13 (1–28)	0 (0–0.01)	31 (3–69)	0.01 (0–0.01)	1.14 (0.95–1.33)
40–44	27 (2–57)	0.01 (0–0.02)	66 (6–140)	0.01 (0–0.03)	1.03 (0.92–1.13)
45–49	53 (5–115)	0.02 (0–0.05)	160 (16–348)	0.03 (0–0.07)	1.32 (1.1–1.54)
50–54	111 (11–247)	0.05 (0.01–0.12)	363 (40–781)	0.08 (0.01–0.18)	1.64 (1.51–1.78)
55–59	205 (24–451)	0.11 (0.01–0.24)	695 (87–1515)	0.18 (0.02–0.38)	1.66 (1.59–1.74)
60–64	316 (38–687)	0.2 (0.02–0.43)	1123 (139–2441)	0.35 (0.04–0.76)	1.9 (1.77–2.03)
65–69	393 (41–860)	0.32 (0.03–0.7)	1634 (179–3415)	0.59 (0.06–1.24)	2.04 (1.88–2.21)
70–74	404 (40–846)	0.48 (0.05–1)	1725 (176–3522)	0.84 (0.09–1.71)	1.88 (1.69–2.07)
75–79	385 (39–802)	0.63 (0.06–1.3)	1612 (168–3125)	1.22 (0.13–2.37)	2.17 (1.96–2.38)
80–84	255 (24–561)	0.72 (0.07–1.59)	1392 (139–2966)	1.59 (0.16–3.39)	2.93 (2.68–3.18)
85–89	114 (11–255)	0.76 (0.07–1.69)	811 (80–1751)	1.77 (0.17–3.83)	3.05 (2.88–3.23)
90–94	28 (3–65)	0.66 (0.06–1.52)	311 (29–696)	1.74 (0.16–3.89)	3.35 (3.25–3.45)
95+	4 (0–11)	0.44 (0.04–1.06)	77 (7–182)	1.41 (0.14–3.34)	3.87 (3.78–3.97)
China	631 (64–1331)	0.08 (0.01–0.17)	2038 (220–4105)	0.1 (0.01–0.2)	1.28 (0.96–1.61)
Sex in China					
Female	313 (33–637)	0.08 (0.01–0.16)	960 (97–1997)	0.09 (0.01–0.18)	1.09 (0.75–1.44)
Male	318 (31–690)	0.09 (0.01–0.18)	1077 (116–2291)	0.11 (0.01–0.24)	1.51 (1.17–1.85)
Age groups in China (years)					
25–29	1 (0–3)	0 (0–0)	1 (0–3)	0 (0–0)	−0.08 (−0.76 to 0.61)
30–34	3 (0–7)	0 (0–0.01)	5 (0–11)	0 (0–0.01)	0.13 (−0.42 to 0.67)
35–39	7 (1–16)	0.01 (0–0.02)	10 (1–24)	0.01 (0–0.02)	0.43 (0.17–0.69)
40–44	14 (1–30)	0.02 (0–0.05)	21 (2–43)	0.02 (0–0.05)	0.14 (−0.08 to 0.37)
45–49	24 (2–52)	0.05 (0–0.1)	54 (5–120)	0.05 (0–0.11)	0.43 (0.01–0.86)
50–54	43 (4–95)	0.09 (0.01–0.2)	111 (12–241)	0.09 (0.01–0.2)	0.72 (0.37–1.08)
55–59	71 (7–157)	0.16 (0.02–0.36)	164 (19–355)	0.15 (0.02–0.32)	0.07 (−0.17 to 0.32)
60–64	98 (11–216)	0.28 (0.03–0.61)	206 (25–459)	0.28 (0.03–0.63)	0.65 (0.35–0.96)
65–69	115 (11–234)	0.42 (0.04–0.86)	353 (36–734)	0.46 (0.05–0.96)	0.91 (0.49–1.32)
70–74	110 (11–237)	0.58 (0.06–1.26)	347 (34–725)	0.65 (0.06–1.36)	0.57 (0.22–0.93)
75–79	83 (8–179)	0.73 (0.07–1.57)	296 (32–624)	0.89 (0.1–1.88)	1.18 (0.79–1.58)
80–84	40 (4–86)	0.75 (0.08–1.62)	264 (28–586)	1.34 (0.14–2.96)	3.59 (3–4.18)
85–89	20 (2–43)	1.16 (0.12–2.55)	149 (14–336)	1.57 (0.15–3.53)	2.06 (1.59–2.53)
90–94	3 (0–8)	1.13 (0.11–2.59)	49 (4–113)	1.67 (0.15–3.85)	1.88 (1.43–2.33)
95+	0 (0–0)	0.47 (0.04–1.06)	7 (1–16)	1.04 (0.1–2.48)	3.91 (3.4–4.43)

Abbreviation: EAPC, estimated annual percentage change.

**Table 2:** Burden of metabolic dysfunction–associated steatotic liver disease attributable to high fasting plasma glucose in China and globally: DALY counts and age‐standardized DALY rates in 1990 and 2021, with temporal trends (1990–2021).

	Number of DALY cases (95% UI) in 1990	The age‐standardized DALY rate/100,000 (95% UI) in 1990	Number of DALY cases (95% UI) in 2021	The age‐standardized DALY rate/100,000 (95% UI) in 2021	EAPC (95% CI)
Global	53,929 (5766–111,051)	1.35 (0.14–2.81)	213,481 (23,797–432,139)	2.45 (0.27–4.95)	2.02 (1.9–2.15)
Sex globally					
Female	26,044 (2790–53,815)	1.22 (0.13–2.53)	99,409 (10,793–204,996)	2.14 (0.23–4.42)	1.96 (1.85–2.07)
Male	27,885 (2964–57,827)	1.5 (0.16–3.12)	114,072 (12,761–230,072)	2.8 (0.31–5.59)	2.07 (1.93–2.21)
Age groups globally (years)					
25–29	190 (18–421)	0.04 (0–0.1)	407 (40–930)	0.07 (0.01–0.16)	1.11 (0.82–1.41)
30–34	348 (32–785)	0.09 (0.01–0.2)	891 (86–1975)	0.15 (0.01–0.33)	1.12 (0.81–1.42)
35–39	691 (54–1501)	0.2 (0.02–0.43)	1648 (146–3678)	0.29 (0.03–0.66)	1.14 (0.95–1.33)
40–44	1277 (103–2756)	0.45 (0.04–0.96)	3168 (295–6763)	0.63 (0.06–1.35)	1.02 (0.92–1.13)
45–49	2295 (197–4943)	0.99 (0.08–2.13)	6903 (682–15,060)	1.46 (0.14–3.18)	1.32 (1.1–1.54)
50–54	4284 (421–9485)	2.02 (0.2–4.46)	13,955 (1531–30,041)	3.14 (0.34–6.75)	1.65 (1.51–1.78)
55–59	6933 (795–15,230)	3.74 (0.43–8.22)	23,487 (2944–51,166)	5.94 (0.74–12.93)	1.67 (1.59–1.74)
60–64	9200 (1116–20,006)	5.73 (0.69–12.46)	32,717 (4051–71,052)	10.22 (1.27–22.2)	1.9 (1.78–2.03)
65–69	9651 (1004–21,085)	7.81 (0.81–17.06)	40,133 (4403–84,019)	14.55 (1.6–30.46)	2.05 (1.88–2.22)
70–74	8156 (816–17,076)	9.63 (0.96–20.17)	34,908 (3561–71,274)	16.96 (1.73–34.63)	1.88 (1.69–2.06)
75–79	6232 (625–12,987)	10.12 (1.02–21.1)	26,070 (2724–50,568)	19.77 (2.07–38.34)	2.16 (1.95–2.38)
80–84	3235 (305–7127)	9.14 (0.86–20.15)	17,643 (1756–37,648)	20.14 (2.01–42.99)	2.92 (2.67–3.17)
85–89	1154 (114–2566)	7.63 (0.75–16.98)	8195 (802–17,633)	17.92 (1.75–38.57)	3.05 (2.88–3.22)
90–94	247 (23–573)	5.76 (0.54–13.38)	2728 (255–6100)	15.25 (1.43–34.1)	3.35 (3.25–3.45)
95+	37 (3–89)	3.62 (0.34–8.78)	627 (60–1484)	11.5 (1.11–27.22)	3.82 (3.73–3.91)
China	16,333 (1663–34,613)	1.89 (0.19–3.97)	45,832 (4935–92,455)	2.13 (0.23–4.28)	0.96 (0.66–1.25)
Sex in China					
Female	7666 (812–15,868)	1.76 (0.19–3.64)	20,229 (2091–42,063)	1.8 (0.18–3.73)	0.69 (0.38–1)
Male	8667 (835–18,249)	2.02 (0.2–4.28)	25,603 (2760–55,008)	2.48 (0.26–5.34)	1.21 (0.91–1.51)
Age groups in China (years)					
25–29	90 (8–204)	0.08 (0.01–0.19)	85 (9–196)	0.1 (0.01–0.23)	−0.08 (−0.76 to 0.61)
30–34	176 (14–413)	0.2 (0.02–0.47)	293 (27–666)	0.24 (0.02–0.55)	0.13 (−0.41 to 0.68)
35–39	377 (30–861)	0.41 (0.03–0.94)	540 (50–1288)	0.51 (0.05–1.22)	0.43 (0.17–0.69)
40–44	655 (52–1467)	0.98 (0.08–2.19)	989 (90–2074)	1.08 (0.1–2.27)	0.13 (−0.09 to 0.36)
45–49	1044 (81–2235)	2.02 (0.16–4.33)	2337 (225–5173)	2.12 (0.2–4.69)	0.43 (0.01–0.85)
50–54	1634 (146–3656)	3.42 (0.31–7.66)	4268 (452–9241)	3.53 (0.37–7.65)	0.73 (0.38–1.09)
55–59	2392 (244–5317)	5.52 (0.56–12.26)	5561 (631–12,010)	5.06 (0.57–10.92)	0.08 (−0.17 to 0.33)
60–64	2863 (321–6297)	8.1 (0.91–17.82)	5985 (723–13,348)	8.2 (0.99–18.28)	0.65 (0.35–0.95)
65–69	2809 (266–5717)	10.3 (0.97–20.96)	8637 (882–17,959)	11.26 (1.15–23.41)	0.92 (0.5–1.34)
70–74	2220 (213–4786)	11.8 (1.13–25.43)	7012 (685–14,670)	13.16 (1.29–27.52)	0.57 (0.22–0.93)
75–79	1338 (136–2893)	11.76 (1.19–25.42)	4787 (509–10,074)	14.45 (1.54–30.42)	1.17 (0.78–1.56)
80–84	505 (52–1091)	9.53 (0.97–20.59)	3343 (359–7415)	16.89 (1.82–37.47)	3.57 (2.98–4.17)
85–89	198 (20–436)	11.73 (1.17–25.83)	1510 (141–3396)	15.85 (1.48–35.66)	2.05 (1.59–2.52)
90–94	31 (3–70)	9.95 (0.94–22.79)	431 (39–992)	14.7 (1.34–33.84)	1.88 (1.43–2.32)
95+	2 (0–4)	3.93 (0.32–8.89)	55 (5–131)	8.59 (0.8–20.48)	3.88 (3.36–4.41)

Abbreviations: DALYs, disability‐adjusted life years; EAPC, estimated annual percentage change.

#### 3.1.2. Gender‐Specific Differences

Both global and Chinese data exhibited a higher disease burden in males than in females. Globally, the ASDR for males (0.13 per 100,000) exceeded that for females (0.11 per 100,000). ASDAR values of 2.80 and 2.14 per 100,000 were noted, respectively. In China, the male ASDR is 0.11 per 100,000, and the ASDAR is 2.48 per 100,000, which were also higher than those of females (0.09 and 1.80 per 100,000, respectively) (Tables 1 and 2). A similar sex‐specific pattern was observed in both China and the global population, with consistently higher ASDRs and ASDARs among males. Moreover, the gender disparity in disease burden was more pronounced in China than the global average. The differences between males and females were particularly evident in ASDAR estimates.

#### 3.1.3. Age‐Stratified Characteristics

**Figure 2:**
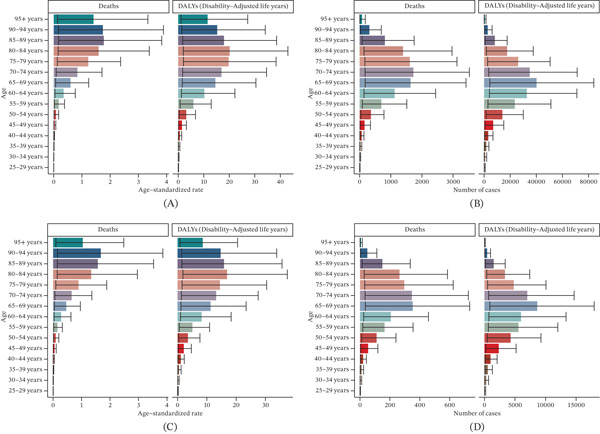
Numbers and ASRs of metabolic dysfunction–associated steatotic liver disease attributable to high fasting plasma glucose‐related deaths and DALYs for different age groups in China and globally in 2021. Abbreviations: DALYs, disability‐adjusted life years; ASRs, age‐standardized rates.

The disease burden, both globally and in China, demonstrates a consistent upward trend with advancing age, primarily affecting the elderly population (Figure 2). In 2021, the Chinese ASDR peak was recorded in the 90–94 years age group (1.67 per 100,000), whereas the global peak occurred slightly earlier in the 85–89 years age group, reaching 1.77 per 100,000. The ASDAR for both regions, China and the world, peaked in the 80–84 years age group (20.14 and 16.89 per 100,000, respectively). Individuals under 50 years of age showed low levels of disease burden, while a marked increase was observed in those aged 60 years and older. In aggregate, age‐specific analyses indicated that the burden of HFPG‐attributable MASLD increased progressively with age in both China and the global population, with the highest burden observed among older age groups.

### 3.2. Temporal Trend Analysis of HFPG‐Attributable MASLD Burden Globally and in China (1990–2021)

#### 3.2.1. Long‐Term Trends and Magnitude of Increase

**Figure 3:**
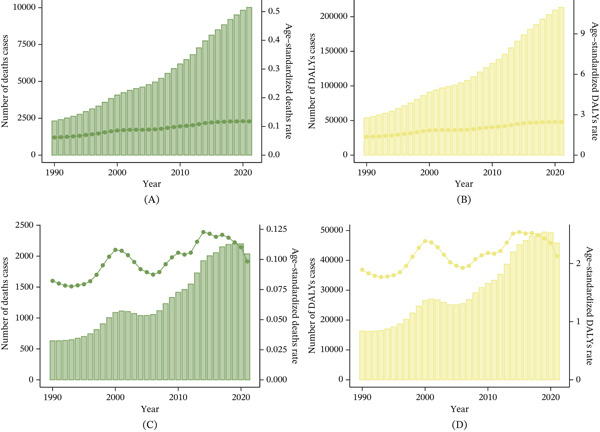
Trends in the numbers and ASRs of metabolic dysfunction–associated steatotic liver disease attributable to high fasting plasma glucose‐related deaths and DALYs in China and globally from 1990 to 2021. Abbreviations: DALYs, disability‐adjusted life years; ASRs, age‐standardized rates; ASDR, age‐standardized death rate; ASDAR, age‐standardized DALY rate.

Between 1990 and 2021, the absolute disease burden of MASLD attributable to HFPG showed a consistent increase both globally and within China (Figure 3). Globally, the number of deaths surged from 2319 to 10,021 (a 332% increase), and DALYs increased from 53,929 to 213,481 (a 296% increase). In China, deaths increased from 631 to 2038 (with a 223% increase), and DALYs grew from 16,333 to 45,832 (with a 181% increase) (Tables 1 and 2). Both global and Chinese estimates demonstrated substantial increases in the absolute burden of HFPG‐attributable MASLD during the study period.

Regarding ASRs, the global ASDR increased from 0.06 to 0.12, and ASDAR rose from 1.35 to 2.45 per 100,000, with corresponding EAPCs of 2.22% (95% CI: 2.07%–2.36%) and 2.02% (95% CI: 1.90%–2.15%), respectively (Tables 1 and 2). In China, the ASDR increased from 0.08 to 0.10 per 100,000, and ASDAR rose from 1.89 to 2.13 per 100,000, with EAPCs of 1.28% and 0.96%, respectively. Although increasing trends were observed in both regions, the EAPCs in China were consistently lower than the corresponding global estimates (Figure 3).

#### 3.2.2. Dynamic Changes Across Gender Dimensions

Throughout the study period, the growth rate of the disease burden was higher in males compared to females, both globally and in China. The EAPC for ASDR in males globally was measured at 2.27%, compared to 2.16% for females. Similarly, in China, the EAPC for ASDR in males (1.51%) was higher compared to females (1.09%). Trends in DALYs were in accordance with these findings (Tables 1 and 2). A persistent sex‐related difference in temporal trends was observed across both global and Chinese populations, with males showing higher EAPCs for both mortality and DALY burden. This disparity was associated with a gradual widening of the gender gap in disease burden over time (Figure 3).

#### 3.2.3. Age‐Specific Growth Characteristics

**Figure 4:**
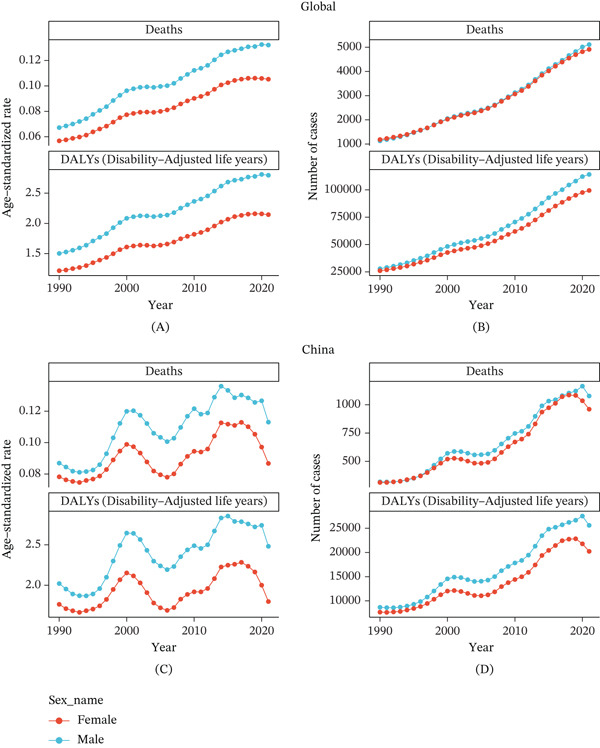
Trends in the numbers and ASRs of metabolic dysfunction–associated steatotic liver disease attributable to high fasting plasma glucose‐related deaths and DALYs in China and globally by sexes from 1990 to 2021. Abbreviations: DALYs, disability‐adjusted life years; ASRs, age‐standardized rates.

Marked variations in the growth of disease burden were observed across different age groups (Figure 4). The increase was relatively slow among young and middle‐aged populations but more pronounced among the elderly. The EAPC for ASDR in individuals aged ≥ 95 years was 3.87% globally and 3.91% in China, surpassing all other age groups (Table 1). Notably, the EAPC for ASDR and ASDAR in the Chinese population aged 25–29 years remained close to zero or demonstrated a slight downward trend (Tables 1 and 2 and Figure 4). Collectively, age‐specific analyses demonstrated that the temporal increase in HFPG‐attributable MASLD burden became progressively greater with advancing age in both China and the global population.

### 3.3. Forecasting of the HFPG‐Attributable MASLD Burden Globally and in China (2022–2046)

#### 3.3.1. Trends in Absolute Disease Burden

**Figure 5:**
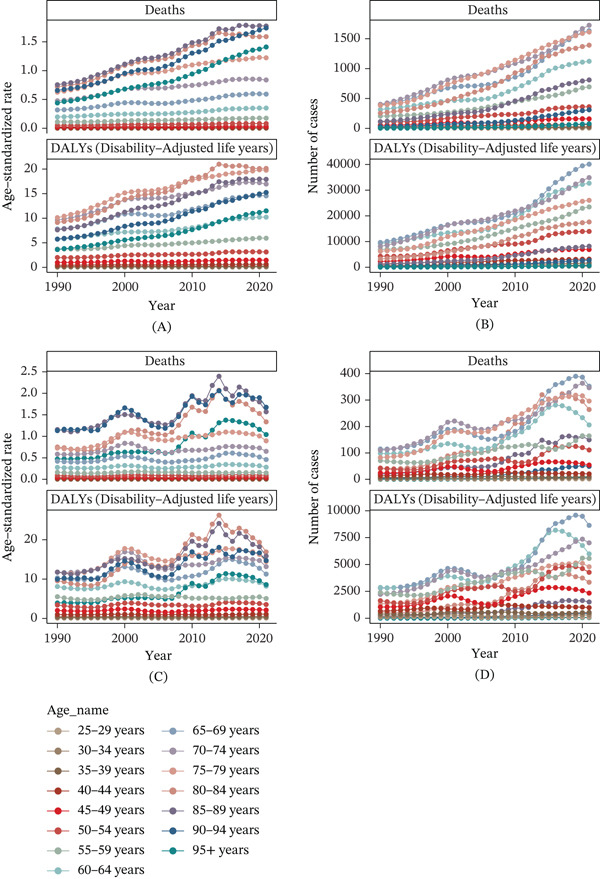
Trends in the numbers and ASRs of metabolic dysfunction–associated steatotic liver disease attributable to high fasting plasma glucose‐related deaths and DALYs in China and globally by age groups from 1990 to 2021. Abbreviations: DALYs, disability‐adjusted life years; ASRs, age‐standardized rates.

Forecasting results predict that both deaths and DALYs from MASLD attributable to HFPG will continue to rise both globally and in China through 2046. Female deaths are projected to increase from 5511 in 2022 to 10,402 by 2046 (with an approximately 89% increase) globally, while male deaths are expected to rise from 5724 to 10,391 (with an approximately 82% increase). In China, female and male deaths are forecasted to grow from 1209 to 1967 (a 63% increase) and from 1374 to 2118 (a 54% increase), respectively. The trends for DALYs are consistent with those of mortality. At a broad level, the projected estimates indicated sustained increases in the absolute burden of HFPG‐attributable MASLD in both China and the global population over the forecast period (Figure 5 and Tables 3 and 4).

**Table 3:** The predicted results in the metabolic dysfunction–associated steatotic liver disease attributable to high fasting plasma glucose‐related numbers and age‐standardized rates of deaths and DALYs by sex globally from 2022 to 2046 of the APC model.

Year	Sex	Number of death cases	The age‐standardized death rate	Number of DALY cases	The age‐standardized DALY rate
2022	Female	5510.887843	0.11	113,028.196	2.36
2023	Female	5750.048772	0.12	118,383.1887	2.41
2024	Female	6005.100724	0.12	123,997.3794	2.45
2025	Female	6232.042974	0.12	129,054.9051	2.48
2026	Female	6466.057948	0.12	134,250.4838	2.51
2027	Female	6704.124228	0.12	139,542.9562	2.54
2028	Female	6951.556796	0.12	144,988.9014	2.56
2029	Female	7213.11289	0.12	150,646.0908	2.59
2030	Female	7437.752779	0.12	155,464.8808	2.61
2031	Female	7665.46094	0.12	160,339.9295	2.62
2032	Female	7892.336261	0.12	165,219.9893	2.63
2033	Female	8123.044107	0.12	170,150.907	2.64
2034	Female	8362.400287	0.12	175,193.2475	2.65
2035	Female	8554.221039	0.12	179,135.9786	2.65
2036	Female	8743.385376	0.12	183,045.9146	2.64
2037	Female	8926.175131	0.12	186,880.9819	2.64
2038	Female	9106.963169	0.12	190,685.3445	2.63
2039	Female	9290.557097	0.12	194,515.847	2.63
2040	Female	9461.983024	0.12	198,052.8177	2.62
2041	Female	9628.68936	0.12	201,532.013	2.61
2042	Female	9787.737226	0.11	204,921.0384	2.6
2043	Female	9943.335259	0.11	208,263.9349	2.59
2044	Female	10,099.6707	0.11	211,609.7228	2.59
2045	Female	10,253.8516	0.11	214,926.5165	2.58
2046	Female	10,402.84344	0.11	218,177.3988	2.57
2022	Male	5723.810232	0.14	128,066.2289	3.05
2023	Male	5975.669807	0.15	133,841.5703	3.1
2024	Male	6243.966819	0.15	139,880.6798	3.16
2025	Male	6479.421993	0.15	145,137.3072	3.19
2026	Male	6720.77578	0.15	150,516.8423	3.22
2027	Male	6964.273084	0.15	155,966.7563	3.26
2028	Male	7215.918895	0.15	161,549.8221	3.29
2029	Male	7481.220417	0.15	167,334.4548	3.32
2030	Male	7699.548362	0.15	172,001.6746	3.33
2031	Male	7919.812665	0.15	176,707.8495	3.34
2032	Male	8137.921163	0.15	181,397.7144	3.35
2033	Male	8359.156663	0.15	186,125.5747	3.36
2034	Male	8589.041868	0.15	190,963.1119	3.37
2035	Male	8760.962495	0.15	194,472.7438	3.36
2036	Male	8930.318122	0.15	197,944.5347	3.34
2037	Male	9093.367083	0.15	201,330.7147	3.33
2038	Male	9255.166023	0.15	204,685.0634	3.32
2039	Male	9420.884437	0.15	208,072.7358	3.3
2040	Male	9570.799182	0.15	211,108.7932	3.28
2041	Male	9716.490719	0.14	214,081.124	3.27
2042	Male	9854.590907	0.14	216,946.3781	3.25
2043	Male	9989.857184	0.14	219,756.576	3.23
2044	Male	10,126.83651	0.14	222,570.0998	3.21
2045	Male	10,261.89119	0.14	225,344.119	3.19
2046	Male	10,391.43318	0.14	228,032.4573	3.17

Abbreviations: APC, age–period–cohort; DALYs, disability‐adjusted life years.

**Table 4:** The predicted results in the metabolic dysfunction–associated steatotic liver disease attributable to high fasting plasma glucose‐related numbers and age‐standardized rates of deaths and DALYs by sex in China from 2022 to 2046 of the APC model.

Year	Sex	Number of death cases	The age‐standardized death rate	Number of DALY cases	The age‐standardized DALY rate
2022	Female	1209.40462	0.11	25,196.40787	2.19
2023	Female	1255.722703	0.11	26,082.34236	2.19
2024	Female	1306.150817	0.11	27,032.50245	2.2
2025	Female	1350.43963	0.11	27,822.75411	2.19
2026	Female	1395.290511	0.11	28,608.34296	2.18
2027	Female	1439.875928	0.11	29,378.19383	2.17
2028	Female	1486.284954	0.11	30,168.61025	2.16
2029	Female	1536.447722	0.11	31,014.15428	2.16
2030	Female	1579.165518	0.1	31,680.48811	2.14
2031	Female	1620.667122	0.1	32,306.46609	2.12
2032	Female	1659.630485	0.1	32,870.11986	2.1
2033	Female	1698.182165	0.1	33,402.82436	2.08
2034	Female	1738.671187	0.1	33,946.67179	2.06
2035	Female	1770.190214	0.1	34,294.25952	2.03
2036	Female	1798.768069	0.1	34,577.03812	2
2037	Female	1823.215794	0.1	34,780.79997	1.97
2038	Female	1845.479935	0.09	34,927.06629	1.94
2039	Female	1868.019822	0.09	35,055.11914	1.91
2040	Female	1889.475589	0.09	35,169.52246	1.89
2041	Female	1907.818144	0.09	35,233.31694	1.86
2042	Female	1922.224575	0.09	35,245.65812	1.83
2043	Female	1934.603873	0.09	35,233.19913	1.81
2044	Female	1947.00477	0.09	35,221.41528	1.78
2045	Female	1958.429817	0.09	35,196.54649	1.76
2046	Female	1967.256052	0.08	35,133.22718	1.73
2022	Male	1373.912358	0.14	30,890.54942	2.97
2023	Male	1428.566916	0.15	31,992.15954	3
2024	Male	1488.716466	0.15	33,167.54413	3.03
2025	Male	1538.248901	0.15	34,095.50176	3.04
2026	Male	1587.247058	0.15	35,007.73995	3.04
2027	Male	1634.514971	0.15	35,891.16007	3.05
2028	Male	1683.39077	0.15	36,792.99632	3.06
2029	Male	1736.712399	0.15	37,754.4452	3.07
2030	Male	1776.632056	0.15	38,431.49761	3.05
2031	Male	1815.249525	0.14	39,068.41962	3.04
2032	Male	1851.211865	0.14	39,643.87468	3.03
2033	Male	1887.43696	0.14	40,196.7339	3.01
2034	Male	1926.741575	0.14	40,773.21034	3
2035	Male	1951.145548	0.14	41,036.82034	2.96
2036	Male	1973.242051	0.14	41,246.8696	2.93
2037	Male	1991.741151	0.14	41,387.97182	2.9
2038	Male	2009.722589	0.13	41,493.01526	2.86
2039	Male	2029.881128	0.13	41,603.49773	2.83
2040	Male	2048.830903	0.13	41,683.18706	2.8
2041	Male	2064.761683	0.13	41,717.54154	2.77
2042	Male	2076.314758	0.12	41,699.3513	2.73
2043	Male	2086.667688	0.12	41,662.42597	2.7
2044	Male	2098.562183	0.12	41,640.32503	2.67
2045	Male	2109.82931	0.12	41,612.20042	2.64
2046	Male	2118.053793	0.12	41,549.23259	2.61

Abbreviations: APC, age–period–cohort; DALYs, disability‐adjusted life years.

#### 3.3.2. Trends in ASRs

**Figure 6:**
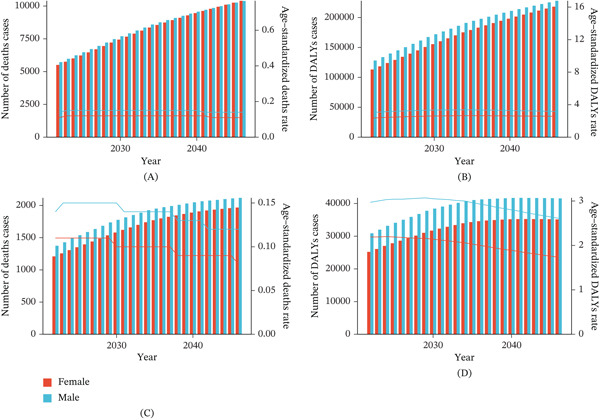
Projections of metabolic dysfunction–associated steatotic liver disease attributable to high fasting plasma glucose deaths and DALYs in China and globally: APC model forecasts by sex, 2022–2046. Abbreviations: DALYs, disability‐adjusted life years; ASDR, age‐standardized death rate; ASDAR, age‐standardized DALY rate; APC, age–period–cohort.

Regarding ASRs, distinct trends are observed globally compared to China. The ASDR and ASDAR are projected worldwide to either fluctuate or remain relatively stable; for instance, the global female ASDR is anticipated to remain at approximately 0.11 per 100,000 between 2022 and 2046, with a similar trend for males. In contrast, China’s ASRs are forecasted to experience a sustained decline. The ASDR for Chinese females is projected to decrease from 0.11 to 0.08 per 100,000 and in males from 0.14 to 0.12 per 100,000; the ASDAR is also anticipated to decrease. These projections suggested differing temporal patterns between China and the global population, with relatively stable ASRs globally but declining ASRs in China (Figure 6 and Tables 3 and 4).

#### 3.3.3. Long‐Term Changes in Gender Disparities

The disease burden is projected to remain consistently higher in males than in females, both globally and within China throughout the forecast period. By 2046, the global ASDAR for males (3.17 per 100,000) is anticipated to exceed that for females (2.57 per 100,000). Similarly, in China, both ASDR and ASDAR will remain elevated in males. Although the decline in ASRs is slightly more pronounced in females, gender disparities are anticipated to persist in the long term. Overall, higher projected ASRs and DALY rates were consistently observed among males in both China and the global population (Figures 5 and 6 and Tables 3 and 4).

## 4. Discussion

Using data from GBD 2021, this study systematically evaluated the burden of MASLD attributable to HFPG at both the global level and in China from 1990 to 2021 and further projected future trends through 2046. Our findings demonstrated a substantial increase in HFPG‐attributable MASLD burden over the past three decades, with marked increases in DALYs. The burden was disproportionately concentrated among males and older adults, highlighting the growing contribution of metabolic risk factors to chronic liver disease worldwide.

A notable finding of this study was the differing epidemiological trends observed between China and the global population. Although both regions experienced increasing burdens, the EAPC of ASDRs in China was lower than the global average. This difference may partially coincide with ongoing improvements in chronic disease prevention and metabolic disease management in China in recent years, including expanded diabetes screening, greater public awareness of metabolic disorders, and the implementation of national public health initiatives such as the “Healthy China 2030” strategy [20, 21]. However, despite projected declines in ASRs, the absolute number of deaths and DALYs in China is expected to continue increasing in the coming decades. This pattern is likely driven by rapid population aging, increasing life expectancy, and the large population size in China [22, 23]. Consequently, the healthcare burden associated with HFPG‐attributable MASLD may continue to increase, particularly among older adults. In addition, substantial regional heterogeneity likely exists across China owing to differences in socioeconomic development, healthcare accessibility, urbanization, and metabolic risk profiles, although such subnational disparities could not be evaluated in the current analysis [11, 24].

HFPG is an important metabolic risk factor associated with IR and metabolic dysfunction, both of which contribute to the development and progression of MASLD [7, 3]. However, MASLD is a multifactorial disease influenced by several interconnected metabolic abnormalities [25, 4]. Obesity, particularly central obesity, dyslipidemia, hypertension, unhealthy dietary patterns, and physical inactivity may interact synergistically with hyperglycemia to increase the risk of hepatic steatosis and adverse liver‐related outcomes [3, 26]. It should be noted that the current GBD CRA framework only permitted quantitative attribution analysis for HFPG in relation to MASLD in this study. Therefore, although other metabolic risk factors are clinically important contributors to disease progression, they were not incorporated into the present attribution model.

The disproportionately high burden observed among older adults may reflect the cumulative effects of long‐term metabolic exposure, multimorbidity, and age‐related physiological decline [27, 28]. Similarly, the higher burden observed among males may be associated with differences in metabolic risk profiles, lifestyle factors, and healthcare utilization patterns [29, 30]. These findings suggest that older adults and men may represent priority populations for early metabolic screening and liver disease prevention strategies [12, 23].

From a clinical and public health perspective, our findings support the importance of integrating liver health assessment into the routine management of patients with diabetes and other metabolic disorders [5, 31]. Early identification of individuals at high risk for MASLD using noninvasive approaches, such as the fibrosis‐4 (FIB‐4) index and vibration‐controlled transient elastography (VCTE), may help improve risk stratification and disease monitoring, particularly in primary care settings [32]. Lifestyle interventions targeting weight reduction, dietary modification, and physical activity remain central components of MASLD prevention and management [33, 34]. Furthermore, emerging pharmacological strategies with dual metabolic benefits, such as GLP‐1 receptor agonists and SGLT2 inhibitors, represent promising avenues for concurrently managing dysglycemia and mitigating hepatic steatosis [35, 36]. In addition, multidisciplinary metabolic management strategies may be particularly important in China, where rapid aging and increasing diabetes prevalence continue to expand the high‐risk population [37, 38].

Several limitations should be considered when interpreting the findings of this study. First, GBD estimates are based on statistical modeling, and the quality and completeness of source data may vary across regions, which could influence the accuracy of burden estimates and long‐term projections. Second, although the nomenclature has shifted from NAFLD to MASLD, GBD 2021 continues to use the historical NAFLD classification; however, previous studies have demonstrated substantial epidemiological overlap between these definitions. Third, due to data limitations, this study could not distinguish between different histological stages of MASLD. In addition, APC‐based projections were generated from historical temporal patterns without direct estimation of forecast UIs. Finally, as an ecological analysis based on population‐level data, this study cannot establish individual‐level causal relationships [39].

Overall, our findings indicate that the burden of HFPG‐attributable MASLD continues to increase globally and remains a growing public health challenge in China. Aging populations and the increasing prevalence of metabolic disorders may further amplify this burden in the future. Strengthening metabolic risk control, improving early screening strategies, and promoting integrated management of metabolic and liver diseases may help reduce the future burden of MASLD [2].

## 5. Conclusion

In conclusion, the global and Chinese burden of MASLD attributable to HFPG increased substantially from 1990 to 2021, with a particularly high burden observed among older adults and males. Although ASRs showed declining or stabilizing trends in some settings, population aging and demographic expansion are projected to contribute to continued increases in the absolute burden through 2046. These findings highlight the importance of strengthening metabolic risk factor prevention and chronic disease management strategies, particularly in rapidly aging populations. However, the projected estimates should be interpreted cautiously because they are based on historical trends and may be influenced by future changes in healthcare systems, population structure, and metabolic risk profiles. Future studies incorporating large‐scale prospective cohort data and longitudinal monitoring are warranted to validate APC‐based projections and further evaluate long‐term associations between glycemic status and liver‐related outcomes across populations.

## Author Contributions

Yong‐Feng Tang had full access to all data in the study and takes responsibility for the integrity of the data and the accuracy of the data analysis. Yong‐Feng Tang was responsible for the conception and design of the study. Xun Wu, Wei‐Wei Zhang, and Bo‐Hu Wang were responsible for the acquisition, analysis, or interpretation of the data. Yong‐Feng Tang drafted and reviewed the manuscript. Xun Wu and Wei‐Wei Zhang made critical revisions to the manuscript for important intellectual content. Bo‐Hu Wang conducted the data analysis. Xun Wu and Wei‐Wei Zhang contributed equally to this study and should be regarded as co‐first authors.

## Funding

The study was funded by the Guangxi Health Commission (self‐funded research project) (No. Z‐A20240967).

## Ethics Statement

Our research involved a secondary evaluation of the publicly accessible GBD study dataset, without primary data collection. Hence, no ethical approval was necessary.

## Consent

No informed consent was required since our research is a secondary analysis of public data.

## Conflicts of Interest

The authors declare no conflicts of interest.

## Supporting Information

Additional supporting information can be found online in the Supporting Information section.

## Supporting information


**Supporting Information 1** Supporting Information Dataset S1 (age.csv). Age‐specific estimates of deaths and DALYs attributable to HFPG‐related MASLD in China and globally from 1990 to 2021.

**Supporting Information 2** Supporting Information Dataset S2 (sex.csv). Sex‐specific estimates of deaths and DALYs attributable to HFPG‐related MASLD in China and globally from 1990 to 2021.

**Supporting Information 3** Supporting Information Dataset S3 (global.csv). Global burden estimates of HFPG‐related MASLD, including deaths, DALYs, age‐standardized death rates (ASDRs), and age‐standardized DALY rates (ASDARs) from 1990 to 2021.

**Supporting Information 4** Supporting Information Dataset S4 (predict1.csv). Projected deaths, DALYs, ASDRs, and ASDARs attributable to HFPG‐related MASLD from 2022 to 2046 based on the age–period–cohort (APC) model.

**Supporting Information 5** Supporting Information Dataset S5 The authors have read the STROBE Statement—checklist of items, and the manuscript was prepared and revised according to the STROBE Statement—checklist of items.

## Data Availability

The data were obtained from a public database and can be accessed through the following link for the relevant data: https://vizhub.healthdata.org/gbd-results/.
